# Genome sequences of four A1 subcluster *Mycobacterium smegmatis* bacteriophages

**DOI:** 10.1128/mra.01331-24

**Published:** 2025-03-25

**Authors:** Spencer T. Payne, Jayden S. Longhurst, Elisa A. Correa Lazaro, Matthew N. Jackson, Jacob D. Scott, Sophie B. Daines, Cade B. Brink, Hayzen H. Chamberlain, Jacob D. Gwilliam, George Higgins, Monterey D. Domike, Rachel E. Moffat, Abbey R. Larson, Payson C. Danielson, Hyunbi Hwang, Shule M. Aggabao, Harry M. Peless, Atalie B. Bogh, Kyla Radke, Bartel Van Oostendorp, Christopher C. Harrell, Austin M. Johnson, Natalie A. Olsen, Parker Danielson, Thomas Wilhite, Jeffrey K. Schachterle, Staci Avery, Donald P. Breakwell, Brett E. Pickett

**Affiliations:** 1 Department of Microbiology, Brigham Young University 6756, Provo, Utah, USA; Portland State University, Portland, Oregon, USA

**Keywords:** Cluster A1

## Abstract

Payneful, Marchy, Hami1, and Sorpresa are A1 subcluster tailed bacteriophages belonging to the *Caudoviricetes* class that infect *Mycobacterium smegmatis* strain mc^2^155. They are consistent with other A1 subcluster phages based on their genome length and guanine–cytosine content. Their genomes contain six novel open reading frames.

## ANNOUNCEMENT

Bacteriophages continue to be a substantial source of advancements in molecular biology, ranging from host restriction enzymes to CRISPR-Cas systems (1). Additionally, mycobacteriophages have the potential to be used as therapeutics, particularly as an alternative to antimicrobial agents, due to their ability to infect mycobacterial species (2).

**Fig 1:**
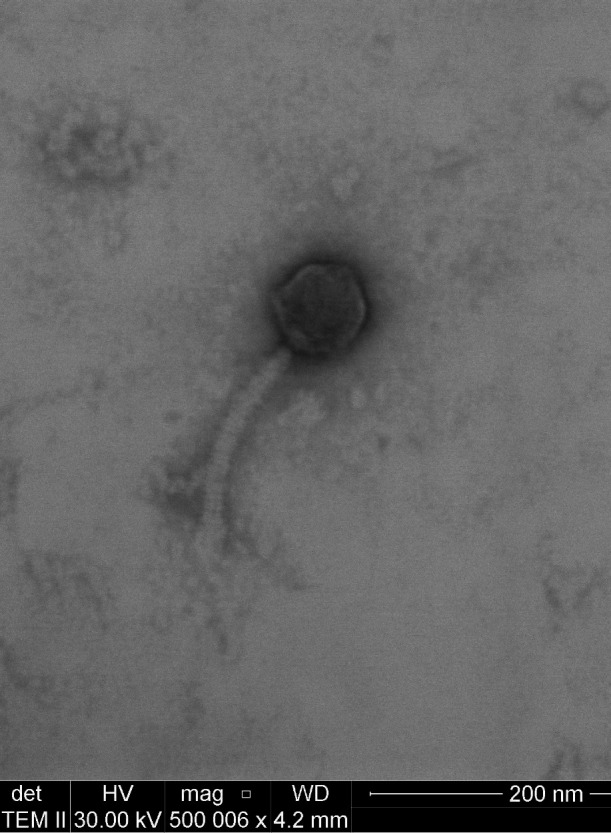
Negative-stained transmission electron micrograph of the Payneful phage (500,000× magnification, 30 kV accelerating voltage, Tecnai TF-20) representative of these four phages.

The phages reported in this study were isolated from soil samples collected in Provo, Utah. Each sample was suspended in 7H9 broth and filtered through a 0.45 µm filter. An aliquot was used to infect *Mycobacterium smegmatis* mc^2^155 host cells mixed with top agar, then plated on 7H10 agar prior to incubation at 37°C for 2 days. Resulting plaques (ranging from 2.5 to 4.0 mm with cleared centers and cloudy outer edges) were picked using a sterile micropipette tip. Following at least four rounds of plaque purification, a high titer lysate (>1E10^9^ PFU/mL) was prepared by flooding nearly confluent “web-plates” with Middlebrook 7H9 broth, incubating for 2 h at room temperature, decanting, and filtering through a 0.2 µm filter. DNA was extracted from the lysate by using the Norgen Phage DNA Isolation Kit following the manufacturer’s protocol prior to library preparation and sequencing. Briefly, 0.5 µg of DNA per sample was used for DNA library preparation using the NEBNext Ultra DNA Library Prep Kit for Illumina (NEB, USA) following manufacturer’s recommendations, and unique indices were added to each sample. The DNA samples were sonicated to a size of 350 bp, then DNA fragments were end-polished, A-tailed, and ligated with the full-length adaptor for Illumina sequencing with further PCR amplification. PCR products were purified (AMPure XP System) and libraries were analyzed for size distribution with an Agilent 2100 Bioanalyzer and quantified using real-time PCR. The whole genomes were sequenced using Illumina NovaSeq X PE150 with the corresponding sequencing kit, producing 2.5–3.5 million 150 bp paired-end reads per genome (Table 1). Reads were trimmed using TrimGalore v0.6.6 (3) with a minimum length of 20 bases and a minimum Phred quality score of 20. Trimmed reads underwent assembly with either Unicycler version 0.5.0 (4) or Newbler v2.9 (5) prior to validation using CONSED v2.9 (6) with default parameters. Phages were visualized using negatively stained (2% uranyl acetate) transmission electron micrograph (Fig. 1).

**TABLE 1:** Features of the four A1 subcluster mycobacteriophages

Phage name	Hami1	Marchy	Payneful	Sorpresa
GPS coordinates	40.47223 N, 111.78163 W	40.262447 N, 111.65287 W	40.24018 N, 111.657454 W	40.27669 N, 111.645 W
Isolation details (year, temperature, depth)	2023 15°C Surface	2023 11°C Surface	2024 34°C Surface	2023 14°C Surface
Sequence reads (millions)	2.62	3.31	3.41	2.52
Sequence depth (× coverage)	2,654	4,941	4,807	3,642
Genome length (base pairs)	47,823	50,273	49,415	52,066
Guanine–cytosine content (%)	63.5	62.7	63.8	63.2
Number of ORFs	92	87	85	93
Number of Orphams	3	1	1	1
Number of ORFs with putative function	41 (45%)	41 (47%)	39 (46%)	38 (41%)
Number of ORFs with no putative function	51 (55%)	46 (53%)	46 (54%)	55 (59%)

Assembled fasta files were loaded into DNA Master v5.23.6 (7) where ORFs were called with data generated by Glimmer (8) and GeneMark (9), BLASTN (10), HHpred (11, 12), GeneMarkS (13), Starterator (14), and Phamerator (15). Functional assignments were made by comparison to the same BLAST and HHpred hits, as well as to the Starterator and Phamerator maps (15). Default parameters were used for all tools. The same tools were used to determine the presence of coding regions in large gaps between ORFs for ORFs missed by automated annotation, and in some cases, ORFs were manually added. tRNA analysis was performed using PhageScope (16) and tRNAscan-SE (17). These genomes were assigned to cluster A1 through best-match nucleotide sequence homology searches (95.94% identity, 84% coverage with A1 phage Maroc7).

Six orphams defined as no BLAST hit with e-values < 1e−5 were found across these four phages. The guanine–cytosine content for the phages was consistent with that of their host and other A1 subcluster phages, ranging from 62.7 to 63.8%. No tRNAs were found in these genomes, although ORF 68 of Sorpresa appeared to encode for a putative transposase.

## Data Availability

Payneful, Marchy, Hami1, and Sorpresa available in GenBank with accession numbers PQ362659, PQ362668, PQ362672, and PQ184814, respectively, and in the Sequence Read Archive (SRA) with accession numbers SRX27013416, SRX27013415, SRX27013412, and SRX27013407, respectively.
